# Folic Acid Supplementation for Women of Childbearing Age versus Supplementation for the General Population: A Review of the Known Advantages and Risks

**DOI:** 10.1155/2011/173705

**Published:** 2011-05-23

**Authors:** Nikhil Shelke, Louis Keith

**Affiliations:** ^1^College of Arts and Sciences, Loyola University, Chicago, IL 60660, USA; ^2^Department of Obstetrics and Gynecology, Northwestern University, Chicago, IL 60611, USA

## Abstract

This paper focuses on the current best-evidence-based clinical practices and controversies surrounding folic acid supplementation/fortification for the prevention of neural tube defects (NTDs) during early pregnancy. The paper also discusses the controversies surrounding the effect of folic acid on the prevention as well as the promotion of cancer. Sufficient data is available to safely conclude that folic acid reduces the risk of NTDs during pregnancy; however, a safe dosage has not yet been calculated for the rest of the population. More research is necessary to study the complete role of folic acid in human growth and development.

## 1. Folic Acid Supplementation Today

Neural tube defects (NTDs) are common and devastating congenital malformations of the central nervous system. Over 90% of the cases are comprised of anencephaly (a total or partial absence of brain tissue, skull, and overlying skin) and spina bifida (herniation of the spinal cord, meninges, or both through a defect in the spinal column). Both arise from incomplete closure of the neural tube early in gestation, before 28 days after conception, and often before a woman is aware that she might be pregnant [1]. NTDs are a worldwide problem, with approximately 300,000 affected newborns every year and 3,000 cases per year in the United States alone [2]. A 2005 report from the Centers for Disease Control (CDC) estimated that the rates for spina bifida and anencephaly were 17.96 and 11.11 per 100,000 live births, respectively, in the United States [3]. In 1992, the FDA recommended that every woman of childbearing age should consume 0.4 mg folic acid daily [4]. This 0.4 mg dose was based on several case control and cohort studies as well as a 1992 Hungarian randomized controlled trial (RCT) that used 0.8 mg daily for primary prevention [5]. In 1999, Berry et al. provided additional evidence for 0.4 mg supplementation when a study carried out in China showed significant primary prevention of NTDs with that dose [6, 7]. 

The protective effect of folic acid delivered during the periconceptual period is the best studied of the numerous potential environmental factors contributing to NTDs. Folate is a water-soluble B vitamin that acts as a cofactor in one-carbon transfer reactions and plays a central role in nucleic acid biosynthesis [8]. Emerging research shows that folic acid may play dual and opposite roles in relation to cancer promotion [9–11]. Because folate deficiency has long been suspected to promote cancer through destabilization of the genome, possibly through increased homocysteine levels, decreased methylation of the genome, uracil misincorporation, and double-strand breaks of DNA leading to chromosomal damage [12, 13], adequate levels of folate could be protective against carcinogenesis through stabilization of the genome. However, because of the critical role that folate plays in nucleic acid biosynthesis, it may also have a role in cancer promotion in susceptible individuals. In patients harboring preneoplastic and neoplastic lesions, exposure to extra folate (through supplementation or fortification) could potentially promote cellular proliferation and cancer growth [10, 14]. This is particularly true for those individuals who already possess tissues which have undergone early changes characteristic of malignancy. For example, in a standard chemical carcinogen rodent model of colorectal cancer, extreme dosages of folic acid (>20-times the basal daily dietary requirement) have been shown to increase the development and progression of colorectal cancer. On the other hand, two other rodent models have shown that modest levels of folic acid supplementation (4–10-times the basal daily dietary requirement) suppressed the development and progression of colorectal cancer. Both, animal studies and recent epidemiological studies have begun to challenge the widely accepted notion that folate status is inversely related to the risk of developing cancers [14]. Studies have shown conflicting or equivocal results for the role of folic acid in other cancers, including breast [15, 16], prostate [17], and neuroblastoma cancers [18]. The inability to reach a consensus represents a pressing issue that deserves further and detailed study in view of the fortification programs that are underway throughout the world, potentially exposing billions of people to higher levels of folic acid.

The biochemical pathway of folic acid that works to prevent NTDs is currently unclear, and many questions about the processes underlying normal and abnormal neurulation remain unanswered and the subject of continued investigation [17]. What is well established, however, is that maternal folic acid intake during the periconceptual period is effective in reducing the occurrence of NTDs [19–21]. One of the most impressive studies illustrating this point was undertaken in China in an area of high NTD prevalence. Half the population (geographically confined) was given folic acid supplementation whereas the other half received normal prenatal care. Those mothers who had received supplementation had significantly fewer infants with NTDs [6].

## 2. Controversies in Practice

Unfortunately, putting prevention into practice has been more difficult than initially anticipated, and numerous controversies are present throughout the literature about some of the most important folic acid-related issues facing women and public health authorities worldwide. Among the contentious issues are questions related to the optimal dose of supplemental folic acid (for the prevention of NTDs), the safety of folic acid among individuals of all ages, the risk benefit profiles of synthetic and naturally derived folic acid, the optimal level of fortification, and if this is even an effective strategy, and the root causes of NTDs in different populations. For example, a study on racial and ethnic differences in NTD rates showed that Hispanics had the highest rates of NTD-affected pregnancies, non-Hispanic blacks and Asians the lowest, and the rates of non-Hispanic whites being intermediate between the two [22]. The reason for such disparities is unclear, and the involved factors likely go far beyond inherent folic acid status in different racial/ethnic groups. 

The recommended daily allowance of folic acid has been difficult to assess and remains the subject of much debate. Although many countries have similar recommendations, no international consensus is available on dosage, and some countries do not have official recommendations. In 1992, the United States Food and Drug Administration (FDA) recommended that every woman capable of becoming pregnant should consume 0.4 mg folic acid daily for the prevention of NTDs [4]. Four years later this same governmental agency advised folic acid fortification of enriched grains, such as flour, bread, farina, cornmeal, rice, and pastas [23]. Similar programs were rapidly adopted in many other countries. Despite these advances in fortification, approximately 150 countries, including those of the European Union, have no requirement for fortification, mostly because of safety concerns [24]. Further studies are needed to evaluate the safety of high-dose folic acid in certain populations.

## 3. Folates and Food

The fundamental difference between natural food folates and synthetic folic acid is considerable and largely unappreciated by the medical community and the patients that they treat. Natural food folates are approximately 50% less bioavailable than is the synthetic folic acid used in supplements and fortification. This decreased bioavailability is a result of several factors: (1) the nature of the food matrix, with some folates remaining bound and unavailable within plant material; (2) factors affecting the deconjugation of the polyglutamate form of folates into the monoglutamyl form, which is the form needed for absorption in the small intestine; (3) losses incurred during harvesting, processing, and cooking of foods. Good sources of folate that are often cited include legumes, orange juice, leafy green vegetables, broccoli, and whole grains. Whereas synthetic folic acid is highly stable, leafy green vegetables and legumes undergo a loss of 50–89% of folate after cooking. For example, broccoli has different folate values depending on whether it is cooked, raw, or frozen. The usual Western diet contains about 0.2 mg natural folate per day. Under these circumstances, the 1992 CDC guideline recommending 0.4 mg per day of folic acid means that a woman would have to consume 1000 *μ*/day of natural food folates to obtain that level, taking into account the bioavailability difference [25]. Such a consumption level is unpractical and underscores the potential value of population wide supplementation programs. It is axiomatic that such problems would not be confined solely to women, and that men would find similar difficulties if they tried to rely on diet alone for their daily values.

## 4. Folic Acid and the Question of Malignancy

Sufficient data now exists to suggest that a correlation may be present between the amount of folic acid intake and between the promotion of cancer. Recent evidence indicates that folic acid may facilitate the preliminary stages of specific types of malignancy. Two Norwegian studies, namely, the Norwegian Vitamin Trial and the Western Norway B Vitamin Intervention Trial, found that daily supplementation with 0.8 mg of folic acid and 0.4 mg B12 for more than three years increased the risk of lung cancer by 21%. The two studies measured cancer incidence, cancer mortality, and all-cause mortality in a total of 6837 patients with ischemic heart disease. The results were able to show that treatment with folic acid plus vitamin B12 was associated with increased cancer outcomes and all-cause mortality in patients with ischemic heart disease in Norway, where there is no folic acid fortification of foods. Such studies suggest that high dose folic acid supplementation may unexpectedly increase cancer risks [26]. However, it is important to note that the dose used in these two trials is twice that recommended on an international basis for pregnancy-related intakes. Systematic studies of the safety of high doses of folic acid are lacking, and the absence of data does not imply assurance of safety. At the same time, observational epidemiologic studies show an inverse relationship between folate intake and cardiovascular disease. Several randomized clinical trials have shown that increases in folic acid supplementation led to dramatic decreases in homocysteine levels, which has long been linked to cardiovascular disease [27]. More studies, especially those that focus on the role of folic acid in primary prevention of CV disease and studies that follow cohorts over long periods of time, are required to address some of these issues.

## 5. Comments for Further Consideration

Many questions remain surrounding folic acid, fortification policies, supplementation, and its likely or projected impact on NTDs, public health, and a host of other diseases. Some of the most pressing issues involve recent questions about the role of folic acid in cancer promotion and prevention.

Because of ongoing as well as substantial efforts and collaboration of scientists, public health authorities, nonprofit groups, and governmental agencies, women in many areas of the world benefit from significant improvements in their folic acid status through the use of folic acid supplementation and fortification, alone or combined. Such efforts are likely to continue, especially those with a focus on public health strategies that target population-specific barriers to supplement use. 

This review focused on the effect of folic acid intake and supplementation (Table 1) in women, but recent evidence also suggests that it may be of great importance in men, not only in terms of potential cancer and/or other associated risks, but also in terms of improving sperm quality in men who are partners to women who are unable to conceive. In particular, men who take supplements containing folic acid have improved sperm counts, motility, and decreased numbers of abnormal forms [28]. The number of such individuals is substantial, and many live in countries with few or incomplete medical resources. In such circumstances, use of vitamin supplements may bring cost-effective opportunities for parenthood.

## Figures and Tables

**Table 1:** Advantages and Disadvantages of Folic Acid Supplementation.

Advantages	Disadvantages
When taken before and during pregnancy, reduces numbers of neural tube defects in newborns [6, 7]	Possible increased risk of lung cancer among smokers with high dose folic acid intake [26]
When taken during pregnancy, reduces number of heart defects in newborns, especially ventricular-septal and conotruncal defects [20]	Increased risk of colorectal cancer, and other cancers with high dose folic acid [14]
When taken by “subfertile men,” increases spermatic parameters of absolute numbers and percent motility, while decreasing the percentage of abnormal forms [28]	

Adapted from: [29].
